# Quantum Chemical Study on the Temperature Dependence of Separation of Molecular Hydrogen and Deuterium Using Adsorption on Mn Dihydrogen Complexes

**DOI:** 10.3390/molecules31040636

**Published:** 2026-02-12

**Authors:** Hao Xue, Naoki Kishimoto, Shinya Takaishi

**Affiliations:** Department of Chemistry, Graduate School of Science, Tohoku University, 6-3 Aramaki Aza-Aoba, Aoba-ku, Sendai 980-8578, Japan; xue.hao.p2@dc.tohoku.ac.jp (H.X.); takaishi@tohoku.ac.jp (S.T.)

**Keywords:** quantum chemical calculation, density functional theory, dihydrogen metal complex, Gibbs energy, equilibrium isotope effect

## Abstract

Molecular hydrogen is considered an ideal next-generation energy carrier. There are two methods of hydrogen molecule adsorption: chemical adsorption and physical adsorption. Since chemical adsorption is strong and physical adsorption is weak, an intermediate adsorption mode is necessary to achieve reversible adsorption and desorption at room temperature. In this study, quantum chemical calculations were used to investigate a solid-phase manganese hydrogen complex, [Mn(CO)(dppe)_2_-H_2_]^+^ (referred to as Mn1, and dppe = 1,2-bis(diphenylphosphino)ethane), to determine whether reversible adsorption and desorption at temperature relatively close to room temperature is feasible. Furthermore, since the adsorption energy for D_2_ is not the same as that for H_2_, the feasibility of separating D_2_ and H_2_ was explored by Gibbs energy calculations at different temperatures using the density functional theory. Based on adsorption measurements conducted at 310–365 K, the D_2_/H_2_ separation factor for Mn1 ranged from 2 to 1.5 as observed in our previous study. The results calculated using the M06-2X functional indicated that the D_2_/H_2_ separation factor for Mn1 at 298 K was approximately 2.55, which is superior to the results obtained using the B3LYP and CAM-B3LYP functionals. The isotope separation ability of [Mn(CO)_3_(PCy_3_)_2_-H_2_]^+^ (referred to as Mn2) is slightly inferior to that of Mn1; however, it has an advantage of lower adsorption enthalpy compared to Mn1, making it more suitable for desorption at lower temperatures.

## 1. Introduction

Hydrogen has drawn interest as an ideal energy carrier because of its ability to combust without emitting carbon dioxide, its ability to be converted into electrical energy via fuel cells, and its high gravimetric energy density. However, hydrogen storage and transportation pose safety challenges because hydrogen is explosive and forms weak intermolecular interactions. Additionally, hydrogen can cause metal deterioration via a process known as hydrogen embrittlement; thus, there are numerous challenges that must be addressed before hydrogen can be used practically. The interaction of hydrogen molecules with material surfaces involves either chemisorption, which requires the dissociation of a strong bond that complicates desorption, or physical adsorption, as observed in metal–organic frameworks (MOFs) [1,2,3,4,5], which does not require bond dissociation but has a weak adsorption force at room temperature. Consequently, materials with either weak or strong interactions with hydrogen are not useful for hydrogen storage because of the substantial energy required for adsorption and desorption.

Dihydrogen complexes, in which hydrogen molecules coordinate with metals, facilitate reversible adsorption and desorption at room temperature because the interaction energies are intermediate between those of chemisorption and physisorption. In addition, the electronic interactions of metal complexes with hydrogen are similar to those with deuterium; however, their differing masses result in distinct vibrational frequencies and energy levels, leading to a significant entropy difference at high temperatures. Deuterium is an important isotope in research, used in NMR solvents [6] and in the semiconductor industry [7,8,9,10,11,12,13,14,15,16,17]. Therefore, methods for the efficient room-temperature isotopic separation of hydrogen and deuterium are highly sought after. Currently, the industry generally uses physical isotope separation methods such as cryogenic distillation or Girdler sulfide electrolysis to produce heavy water to produce deuterium and its compounds. These methods [18,19] are time-consuming, energy-intensive, and have low selectivity, necessitating the development of alternatives to these energy-intensive methods. Kinetic quantum sieving (KQS) [20,21,22,23] describes the effect of lighter isotopes with larger de Broglie wavelengths encountering higher energy barriers when diffusing through pores at low temperatures. However, achieving a balance between selectivity and adsorption capacity when separating isotopes using this method is challenging, as pore size restriction increases gas separation selectivity while simultaneously reducing adsorption capacity. Furthermore, this method requires cryogenic separation conditions, which consumes significant amounts of energy. Therefore, we considered using manganese dihydrogen complexes, hydrogen adsorbents with high hydrogen adsorption enthalpy and simple structure, to achieve relatively near-ambient temperature hydrogen isotope separation.

In this study, we estimated the equilibrium temperature for the reversible adsorption and dissociation of hydrogen molecules from dihydrogen complexes at room temperature using density functional theory (DFT) calculations and assessed the isotope separation capabilities (D_2_/H_2_) of these complexes. In other words, the purpose of this study is to qualitatively evaluate the temperature dependence and separation performance of the equilibrium isotope effect of the crystal using DFT calculations of a single model complex molecule. The following quantum chemical calculations were conducted:

1. When the interaction between a hydrogen molecule and the central metal is large, the reaction rate of the desorption reaction becomes small and it takes time to reach chemical equilibrium, which may be a problem that makes the experimental detection difficult. To investigate whether metal complexes are capable of desorbing hydrogen at temperatures close to room temperature, the distances between adsorbed hydrogen atoms in dihydrogen complexes were investigated and correlated with the interactions.
2. Using the artificial force-induced reaction (AFIR) method [24], we calculated the energy needed to separate hydrogen molecules from the central metal of the stable complex. This approach can allow us to estimate the effective energy barrier for the dissociation of M and H_2_. In addition to comparing with experimental values of bond dissociation energy, the calculated effective energy barrier could potentially be used to determine whether the dissociation temperature is high or low.
3. The Gibbs energies of the metal complex molecules interacted with D_2_ or H_2_ were calculated at various temperatures using quantum chemical computations, and the possibility of selectively separating H_2_ and D_2_ was evaluated by the equilibrium constant ratio (*K*_D_/*K*_H_).

## 2. Results and Discussion

### 2.1. H-H Distance and Interaction in Dihydrogen Complexes

As an example of a metal complex that strongly interacts with H_2_, [TaCp_2_(H_2_)(CO)]^+^ was optimized using various theoretical methods (see Table 1). Initially, the B3LYP [25,26] hybrid functional and LanL2DZ basis set were employed because B3LYP is a well-known, robust hybrid functional and LanL2DZ is frequently used in studies of transition-metal complexes. However, a significant discrepancy was noted between the calculated H-H distance (0.876 Å) and the experimental values (0.96–0.98 Å) [27]. Subsequent trials involving the CEP-121G (Stevens/Basch/Krauss ECP triple-split basis) for all atoms or 6-31++G** for hydrogen atoms did not correct this discrepancy in the H-H bond length, which was 84–90% of the experimental value. On changing to the ωB97XD [28] functional, which has long-range correction, the calculated H-H distance approached 91–96% of the experimental value. The longest H-H distance (0.920 Å) was achieved using the LanL2TZ(f) basis set for Ta and other atoms and 6-311++G** for hydrogen atoms. Although LanL2DZ is commonly used for transition-metal calculations, LanL2TZ(f) is anticipated to yield more precise results. The use of the ωB97XD hybrid functional, which includes weak interactions via Grimme’s DFT-D2 dispersion correction, as well as long-range correction, enhanced the agreement between the calculated and experimental H-H distances. It is more advantageous than B3LYP for handling weak interactions in complex systems. Consequently, the ωB97XD functional combined with the LanL2TZ(f) basis set for metal atoms, 6-311++G** basis set for hydrogen atoms, and 6-31+G* basis set for other atoms was applied to [TaCp_2_(CO)(H_2_)]^+^ and Cr(CO)_3_(P/Pr_3_)_2_(H_2_).

Next, the interaction enthalpy, including zero-point vibrations, and interaction Gibbs free energy at 298 K were calculated. The correlation between the H-H distance and interaction energy in these dihydrogen complexes is shown in Table 2. The interaction between the Ta complex molecule and H_2_ was the strongest, and the H-H distance reached a maximum of 0.920 Å. This distance suggests that the H-H bond remains intact because this value is smaller than the typical dihydride complex value of 1.6 Å. Conversely, the interaction with the Cr complex was weaker, and the H-H distance of 0.820 Å is only slightly larger than that of a free hydrogen molecule (0.74 Å).

For a solid crystal of [Mn(CO)(dppe)_2_-H_2_]^+^ (denoted as Mn1) [29], H_2_ adsorption measurement indicated that the enthalpy value of −50.2 kJ/mol [30]. The interaction enthalpy value at 0 K for the Mn1 complex was calculated to be −40.43 kJ/mol by the ωB97XD functional. The optimized H-H distance of 0.83 Å is similar to the Cr complex, while the enthalpy value of −40.43 kJ/mol for Mn1 is not similar to the Cr complex (−58.77 kJ/mol). It should be noted that another hybrid meta exchange-correlation functional, M06-2X, was found to result in weaker interaction in enthalpy (−31.01 kJ/mol) and H-H distance (0.795 Å), while the optimized H-H distance for Mn1 using the M06 functional was 0.854 Å.

### 2.2. AFIR Calculation for Molecular Hydrogen Desorption

Since it is also important in separation experiments to dissociate hydrogen from the complex by adjusting the temperature, it is undesirable for the dissociation reaction to occur poorly, i.e., for the desorption energy to be too large. For AFIR calculations with GRRM23 [31,32], the ωB97XD functional was first employed for two manganese complexes, [Mn(CO)dppe-H_2_]^+^ (=Mn1) and [Mn(CO)_3_(PCy_3_)_2_-H_2_]^+^ (=Mn2) [33], for comparison. We believe that interactions with groups other than the central metal are also important in determining how easily hydrogen atoms can be desorbed. Estimating binding energy solely from the enthalpy difference before and after hydrogen adsorption makes it challenging to deduce the effective activation barrier for dissociation. Hence, approaches like the one presented in this study are considered crucial.

The calculations were performed while gradually increasing the negative force in fixed numerical increments of −2.5 kJ/mol until the GRRM23 software [32] determined that dissociation had occurred on the basis of the distance between two molecular fragments. The choice of this increment value is a numerical parameter ensuring that the determined dissociation energy barrier is resolved with an uncertainty no greater than 2.5 kJ/mol; it does not carry a specific physical meaning. In this case, the structure was optimized by adding negative energy between the central metal and a hydrogen atom in H_2_. As a result, shown in Table 3, the adsorbed hydrogen molecule dissociated with a minimum energy value of 70.0 kJ/mol for Mn1 using ωB97XD. Similarly, a slightly less negative value of 67.5 kJ/mol was calculated as the minimum energy for Mn2. This AFIR calculation accounts for structural relaxation and the effective dissociation barrier required for hydrogen removal with moving sterically repulsive groups. The computed energy value exceeds the experimentally determined adsorption energy (−50.2 kJ/mol for the Mn1 crystal [30]). Next, we tested a different functional to verify if the dissociation reaction barrier could be reduced. As noted in Section 2.1, the M06-2X functional predicts a weakened interaction between the central metal atom and the hydrogen molecule. Using this functional, the dissociation energy barrier for hydrogen molecule desorption was determined to be 42.5 kJ/mol or less for Mn1. This value is lower than that obtained with ωB97XD and, interestingly, is closer to the experimental Δ*H*. Moreover, for Mn2, the AFIR calculation yielded a significantly negative dissociative energy value of 47.5 kJ/mol, indicating an inversion in the relative energies of Mn1 and Mn2 compared to those determined by ωB97XD. The experimental Δ*H* value for Mn2 (−27.0 kJ/mol [31]) falls between the results of the two Δ*H* values by DFT calculations, M06-2X and ωB97XD. The energy required for dissociation of H_2_ was calculated using the AFIR method, but there was not much difference between Mn1 and Mn2. In the experiments, Mn1 required a higher temperature than Mn2, but it seems difficult to connect this fact directly to the AFIR calculations of H_2_ desorption performed this time.

### 2.3. Interaction Gibbs Free Energies for Mn–Dihydrogen Complexes

A significant difference between the adsorption–desorption equilibrium temperature of M-H_2_ and M-D_2_ suggests the promise of this complex for isotope separation. For Mn1 [29], experimental van’t Hoff plots for the adsorption of H_2_ and D_2_ at 313–363 K yielded enthalpy differences (Δ*H*) of −50.2(6) and −54.4(4) kJ/mol for H_2_ and D_2_, respectively. Additionally, entropy differences (Δ*S*) of −121.7(18) and −129.3(13) J/mol·K for H_2_ and D_2_, respectively, were obtained, which could be correlated with Δ*G* = 0 at approximately 412.9 and 421.0 K for H_2_ and D_2_, respectively, using the relationship Δ*G* = Δ*H* − *T*Δ*S*. The temperature difference is 8.1 K.

**Figure 1:**
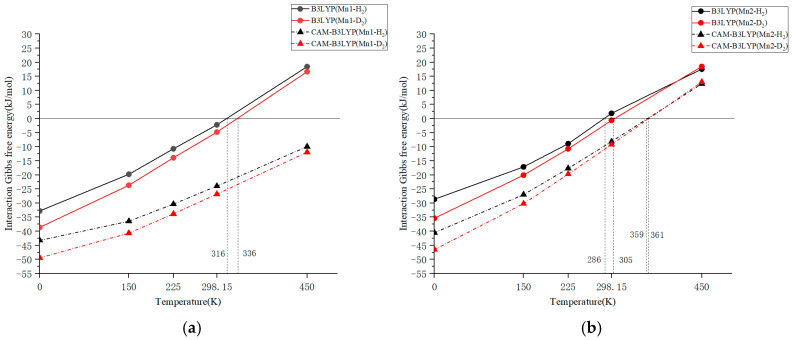
Temperature dependence of Gibbs free energy interaction with H_2_ (∆*G_T_* (H)) or D_2_ (∆*G_T_* (D)) for (**a**) Mn1 and (**b**) Mn2 using B3LYP and CAM-B3LYP functionals.

The temperature dependence of the Gibbs free energy of interaction for Mn1 and Mn2 calculated using the B3LYP functional is depicted in Figure 1a and Figure 1b, respectively. In both figures, the results for H_2_ and D_2_ adsorption are compared, enabling the estimation of the temperature at which the Gibbs free energy of interaction (Δ*G*) becomes zero. The data plotted in the figure is included as a table in the Supporting File. The B3LYP calculations for the molecular model in this study indicated adsorption–desorption equilibrium temperatures of approximately 316 K for H_2_ and 336 K for D_2_, with a temperature difference of ca. 20 K. These theoretical temperatures are approximately 90–100 K lower than the experimental Δ*G* = 0 temperatures, and the estimated Gibbs free energy values for Mn1 at 298.15 K obtained experimentally (Δ*G* = −13.97 kJ/mol for H_2_ and −15.89 kJ/mol for D_2_) are approximately 11 kJ/mol lower than the values calculated using B3LYP (Δ*G* = −2.21 kJ/mol for H_2_ and −4.80 kJ/mol for D_2_).

The table above shows the vibrational entropy in the calculations. It can be seen that when using B3LYP, the changes in vibrational entropy for H_2_ and D_2_ adsorption are minimal. When using other functionals, the vibrational entropy for D_2_ adsorption is larger than that for H_2_ adsorption. This is presumably because other functionals have a more accurate calculation capability for weak interactions.

Since the equilibrium state of adsorption and desorption was observed in experiments conducted within the temperature range of 313–363 K, computational results that show equilibrium within this range are considered favorable. However, the temperature difference between achieving equilibrium with H_2_ and D_2_ may vary depending on the DFT calculations. To refine the Gibbs free energy difference and achieve a quantitative comparison, the temperature dependences of the Gibbs free energy were obtained using various DFT functionals. For the Mn2 complex, the calculated adsorption–desorption equilibrium temperatures were 286 and 305 K for H_2_ and D_2_, respectively. This indicates that H_2_ and D_2_ tend to dissociate more easily at lower temperatures in the case of Mn2. This differs from the AFIR calculation result by M06-2X presented in Section 2.2, and kinetic factors may play a slight role in the temperature dependence of dissociation, alongside thermodynamic control.

The temperature dependence of the Gibbs free energy interactions for Mn1 and Mn2, as determined by CAM-B3LYP—a long-range corrected functional—is also shown in Figure 1a and Figure 1b, respectively. The interaction Gibbs free energy at 0 K (enthalpy) for Mn1 was (−43.24 kJ/mol) for H_2_ adsorption and (−49.45 kJ/mol) for D_2_ adsorption, which are not so far from the experimental Δ*H* values of −50.2(6) kJ/mol for H_2_ and −54.4(4) kJ/mol for D_2_. Due to the increase in entropy of molecular vibrations being very effective in Mn1-H_2_ (or D_2_), the calculated adsorption–desorption equilibrium temperatures of the Gibbs free energy for Mn1 were significantly higher than room temperature: approximately 550 K for H_2_ adsorption and 570 K for D_2_ adsorption (not shown in the figure). These temperatures are also far from the experimentally determined Δ*G* = 0 temperature, making them unlikely to be suitable for Gibbs energy calculations. For the Mn2 complex, the calculated adsorption–desorption equilibrium temperatures were 352 and 361 K for H_2_ and D_2_, respectively. These temperatures are about 60–70 K higher than those calculated using B3LYP, resulting in hydrogen desorption being less likely to occur by the long-range correction.

**Figure 2:**
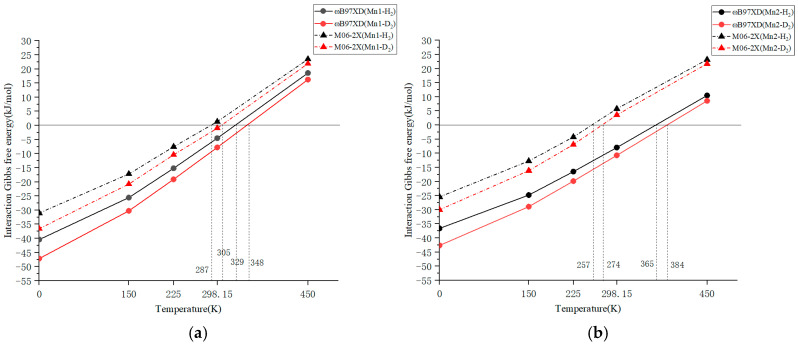
Temperature dependence of Gibbs free energy interaction with H_2_ (∆*G_T_* (H)) or D_2_ (∆*G_T_* (D)) for (**a**) Mn1 and (**b**) Mn2 using ωB97XD and M06-2X functionals.

The ωB97XD calculation results of Mn1 and Mn2 for the interaction with H_2_ are shown in Figure 2a and Figure 2b, respectively. The data plotted in the figure is included as a table in the Supporting File. The adsorption–desorption equilibrium temperature for Mn1-H_2_ will be approximately 329 K and the Gibbs free energy value for Mn1 at 298.15 K is Δ*G* = −4.51 kJ/mol. These results are between B3LYP and CAM-B3LYP and are not far from the experimentally derived Δ*G* = 0 temperature of 412.9 K. Regarding Mn2, the equilibrium temperature is 365 K, and the Gibbs free energy value at 298.15 K is Δ*G* = −7.95 kJ/mol. Based on the temperature dependence of Gibbs energy calculated using ωB97XD in Figure 2, Mn1 appears more suited to lower temperatures compared to Mn2. However, in practice, experiments were conducted at lower temperatures with Mn2 instead. As discussed in Section 2.1 regarding the H-H distance, ωB97XD might overestimate interactions in quantum chemical calculations of manganese dihydrogen complexes. Therefore, we concluded that it is worthwhile calculating the temperature dependence of Gibbs energy using a weaker functional (M06-2X).

Our computational model was further scrutinized by performing quantum chemical calculations using another functional, M06-2X. The temperature dependences of the Gibbs free energy interactions for Mn1 and Mn2 by M06-2X are also shown in Figure 2a and Figure 2b, respectively. The enthalpy difference with H_2_ at 0 K was −31.01 kJ/mol for Mn1 and −25.45 kJ/mol for Mn2, and the calculated Gibbs free energy differences at 298.15 K were +1.34 kJ/mol for Mn1 and +5.77 kJ/mol for Mn2. The temperature at which the Gibbs free energy difference for Mn1 became zero was estimated to be 287 K, whereas the temperature at which Δ*G* = 0 was determined to be 412.9 K for H_2_ adsorption experiment. Thus, a difference of approximately 126 K (=412.9 − 287) remained between the experimental and calculated results using M06-2X. However, the H_2_/D_2_ separation experiments for Mn1 were performed at the temperature range of 313–363 K. From the perspective of the experimental temperatures, ωB97XD can be considered too high, while M06-2X is too low. In addition, it should be noted that the difference between the experimental and computational results may be attributed to the differences between the solid crystal and single-molecule models. This deviation may stem from the gas-phase single-molecule model we used, which failed to account for the crystal field effect and intermolecular interactions present in the experimental solid crystal. In the case of D_2_ adsorption, the calculated Gibbs free energy difference at 298.15 K was −0.98 kJ/mol for Mn1 and +3.60 kJ/mol for Mn2. The temperatures at which Δ*G* = 0 for Mn1 were estimated to be 287 and 305 K for H_2_ and D_2_ adsorption, respectively. For the Mn2 complex, the temperatures at which Δ*G* = 0 were calculated to be 257 and 274 K for H_2_ and D_2_ adsorption, respectively. The calculated adsorption–desorption equilibrium temperature differences between H_2_ and D_2_ adsorption were 18 K for Mn1 and 17 K (=274 − 257 K) for Mn2. This temperature difference indicates that Mn1 is almost same in hydrogen isotope separation than Mn2. However, the adsorption–desorption equilibrium temperature difference estimated from the hydrogen adsorption experiment for Mn1 is 8.1 K, indicating persisting problems with calculation accuracy. Therefore, the isotope separation performance of the compounds was compared using separation factors.

### 2.4. Comparison of Isotope Separation Coefficients for Mn Complexes

Next, we evaluated the calculated isotope separation coefficients (*K*_D2_/*K*_H2_) at the temperatures of 150, 225, 298.15 and 450 K for Mn1 and Mn2 using the B3LYP, CAM-B3LYP, ωB97XD and M06-2X functionals (refer to Table 4), where *K_T_* = exp(−∆*G*/*RT*). Based on adsorption/desorption measurements conducted at 313–363 K, the *K*_D2_/*K*_H2_ ratio for Mn1 was determined to decrease from 1.96 to 1.58 [30]. The calculation results using B3LYP, CAM-B3LYP, and ωB97XD fall outside this range, showing higher coefficient values. At lower temperatures, the separation efficiency increases due to a significant Gibbs energy difference. However, if detecting the separated hydrogen molecules in experiments proves challenging, such temperatures cannot be considered practical.

Regarding Mn2, as mentioned in Section 2.2, Mn2 exhibits a lower experimental adsorption enthalpy (−27.0 kJ/mol) compared to Mn1 (−50.2 kJ/mol). This lower binding strength thermodynamically favors desorption at lower temperatures, which is consistent with experimental observations. For instance, effective chromatographic separation of H_2_/D_2_ using Mn2 has been demonstrated at temperatures of least 213 K, with a separation factor (KD_2_/KH_2_) ranging from 4.2 to 2.2 in the temperature range of 213–273 K [31]. This indicates that Mn2 can operate efficiently as an adsorbent within this experimentally accessible, lower temperature window. Adsorption/desorption measurements conducted at 213–273 K, the *K*_D2_/*K*_H2_ ratio for Mn2 was determined to decrease from 4.2 to 2.2 [31]. Comparing the results (Table 5) of the two DFT calculations, ωB97XD and M06-2X, with the experimentally determined separation coefficient for Mn2, the results from M06-2X deviate less than those from ωB97XD. This also holds true for Mn1.

## 3. Calculation Methods

The relationship between the H-H internuclear distance and bonding energy in the dihydrogen complexes was examined using DFT. All DFT calculations in this study were performed on gas-phase, single-molecule models. No lattice parameters or periodic boundary conditions were applied during geometry optimizations. These calculations were aimed at identifying metal complexes with standard H-H bond lengths as candidates for reversible hydrogen storage at ambient temperatures. This is because if, upon bonding with a complex, the H-H bond length increases and a stable dihydride complex is formed, the complex is unsuitable for reversible hydrogen storage at room temperature. In this study, we optimized the structures of five dihydrogen complexes: [TaCp_2_(CO)H_2_)]^+^, Cr(CO)_3_(P/Pr_3_)_2_H_2_, and [Mn(CO)(dppe)_2_H_2_]^+^. Among these, the H-H distance only in the Ta complex was comparable with the experimental value obtained via solution NMR [27].

The AFIR method [24], as part of the Global Reaction Route Mapping (GRRM) algorithm, can be used to investigate processes by which reactants transition from a state of separation to reaction upon approach. In this method, reactants A and B are initially separated, and an artificial force is applied to push them together. By introducing an additional term—proportional to the distance (*r*_AB_) between A and B—into the energy (*E*), the potential energy curve is modified to *F*(*r*_AB_) = *E*(*r*_AB_) + *αr*_AB_, where *α* is a proportionality constant. The modification enables A and B to bond once they overcome the energy barrier (activation energy) required for the reaction. If *α* is then set to zero, the original potential is obtained, i.e., that without the artificial force. In this study, the activation energy for the dissociation of the bonded hydrogen molecule was estimated roughly by using the AFIR method by introducing negative energy and identifying the maximum energy threshold that prevents dissociation. For this AFIR calculation with GRRM23 [32], the ωB97XD functional was first employed with the following basis sets: LanL2DZ(f) (Los Alamos ECP + DZ) for Mn, aug-cc-pVDZ [34,35] for adsorbing two hydrogen atoms, and 6-311G for other nonmetal atoms. Two manganese complexes, [Mn(CO)dppe-H_2_]^+^ (=Mn1) and [Mn(CO)_3_(PCy_3_)_2_-H_2_]^+^ (=Mn2), were calculated for comparison.

Crucially, identifying a complex that enables the controlled adsorption and desorption of molecular hydrogen at ambient temperature would be advantageous. In this context, thermodynamic stability can be evaluated by calculating Gibbs energy, which primarily depends on the entropy associated with intramolecular vibrations. Moreover, the temperature-dependent Gibbs free energy of interaction is expected to differ when hydrogen or deuterium molecules form a complex because of the difference in vibrational frequencies. The equilibrium structure was determined using the structure optimization feature of the GRRM23 program, and the interaction Gibbs free energy (Δ*G_T_*) at temperature *T* was calculated using a vibrational frequency analysis based on the harmonic oscillator approximation for the optimized structure. The Gibbs free energy of interaction was derived using Equations (1) and (2).

∆*G_T_* (H) = *G_T_* (M(+2H)) − *G_T_* (M) − *G_T_* (H_2_) (1)

∆*G_T_* (D) = *G_T_* (M(+2D)) − *G_T_* (M) − *G_T_* (D_2_) (2)

Here, M represents a metal complex and M(+2H) denotes a metal–dihydrogen complex. In this study, we focused on Mn1 and Mn2 dihydrogen complexes and estimated the adsorption–desorption equilibrium temperature at which the Gibbs free energy of interaction became zero. Thus, we investigated the feasibility of adsorption and desorption near room temperature. Lastly, the deuterium/hydrogen isotope separation coefficients, defined as the ratios of adsorption equilibrium constants (*K*_D_/*K*_H_) for the two Mn complexes, were compared using the standard relationship ∆*G_T_* = −*RT* ln*K*, where *R* is the gas constant and *K* is the equilibrium constant for the molecular adsorption model.

The selection of DFT functionals in this study (B3LYP, CAM-B3LYP, ωB97XD, M06-2X) was guided by their widespread usage in computational organometallic chemistry and our primary objective: to perform a systematic comparative assessment of how different classes of functionals predict key properties (H-H distance, adsorption enthalpy, Gibbs energy, and isotope separation factors) for manganese dihydrogen complexes. While this comparative approach reveals important trends and functional sensitivities, we acknowledge several methodological limitations that should be considered when interpreting the results.

First, regarding electronic structure methods, while ωB97XD includes a dispersion correction (DFT-D2), more advanced dispersion schemes (e.g., Grimme’s D3 or D4 corrections) or functionals specifically optimized for transition metals (such as TPSSh, SCAN, or r^2^SCAN) could provide improved accuracy for weakly bound dihydrogen complexes. Notably, the M06-2X functional, although yielding separation factors closest to the experiment in this study, is not parameterized for transition metals and may not fully capture their electronic structure (including possible multi-reference character). Thus, its agreement with experiment here should be viewed as empirically useful but not theoretically rigorous—a point that underscores the pragmatic versus first-principles nature of this screening study.

Second, our thermodynamic analysis relies on the harmonic oscillator approximation for vibrational entropy. For the large, flexible Mn1 and Mn2 complexes—with numerous low-frequency torsional modes from phosphine ligands—anharmonic effects are likely significant. The harmonic treatment may overestimate entropic contributions, affecting the predicted Gibbs free energies and equilibrium temperatures. Future studies could benefit from quasi-harmonic or molecular dynamics approaches to better capture these effects.

Third, and perhaps most impactful, is our use of a gas-phase single-molecule model, which neglects solid-state phenomena present in the experimental crystals: intermolecular interactions, crystal packing, lattice vibrations, and possible cooperative effects. These omissions contribute to the discrepancies between calculated and experimental ΔG = 0 temperatures and absolute energy values. This model was chosen to enable a clear, computationally tractable comparison across multiple functionals, but it necessarily simplifies the real system.

Despite these limitations, the core contribution of this work lies in its comparative framework. By evaluating four common functionals on the same set of complexes, we systematically map how predictions vary with functional choice—highlighting that even widely used methods can yield substantially different thermodynamic estimates. This insight is valuable for guiding future computational screenings and for interpreting DFT results in similar organometallic systems.

We therefore emphasize that this study is exploratory and qualitative in nature, aimed at identifying trends and methodological sensitivities rather than providing quantitatively precise predictions. Future work should build on these findings by employing (1) more advanced dispersion-corrected or meta-GGA functionals validated for transition metals, (2) anharmonic or dynamic entropy treatments, and (3) periodic or embedded-cluster models to better approximate the solid-state environment. Such steps will be essential for achieving predictive accuracy in the computational design of dihydrogen-complex-based separators.

## 4. Conclusions

In this study, we performed a systematic DFT comparison to evaluate the hydrogen adsorption and isotope separation performance of two manganese dihydrogen complexes (Mn1 and Mn2) under near-ambient conditions. The primary goal was to assess how the choice of density functional influences predictions of structural, energetic, and thermodynamic properties relevant to reversible H_2_/D_2_ separation. The main findings are summarized as follows:

1. Functional Dependence of Predicted Properties: Significant variations were observed in the calculated H-H distances, adsorption enthalpies, Gibbs free energies, and isotope separation factors across the four tested functionals (B3LYP, CAM-B3LYP, ωB97XD, and M06-2X). This result highlights the critical role of functional selection in computational studies of organometallic dihydrogen systems. Notably, the M06-2X functional—though not specifically parameterized for transition metals—gave separation factors in closest agreement with experiment, offering a pragmatic screening tool for future exploratory studies.
2. Consistent Trends for Isotope Separation: Regardless of the functional used, Mn1 consistently showed a slightly higher D_2_/H_2_ separation coefficient than Mn2 around 298 K, suggesting its potential advantage for room-temperature isotope enrichment. The lower adsorption enthalpy of Mn2, also consistently predicted, aligns with its experimentally demonstrated operation at lower temperatures.
3. Methodological Insights and Future Directions: This work intentionally employed a gas-phase single-molecule model and a set of common but not fully optimized functionals to clearly expose methodological sensitivities. The observed deviations from experimental data stem partly from known limitations: the absence of advanced dispersion corrections (e.g., D3/D4), the use of the harmonic approximation for entropy, and the neglect of solid-state effects. These limitations, however, do not diminish the comparative value of the study; rather, they provide a clear basis for further refinement. Future investigations should incorporate the following:
  - Functionals with improved dispersion treatments and better suitability for transition metals (e.g., ωB97X-D3, TPSSh-D4, or SCAN);
  - Anharmonic or quasi-harmonic treatments of low-frequency vibrational modes;
  - Periodic or cluster-embedding models to account for crystal-phase interactions.

In summary, this comparative DFT analysis serves as a valuable methodological reference for computational studies of dihydrogen complexes. It underscores the importance of functional benchmarking and model awareness when predicting thermodynamic properties for isotope separation. The insights gained here not only advance the understanding of Mn-based adsorbents but also contribute to the development of more reliable computational protocols for the design of next-generation hydrogen–isotope separation materials.

## Figures and Tables

**Table 1:** The distance between adsorbed hydrogen atoms *R*(H-H) obtained from structural optimization calculations using varied basis sets and DFT functionals.

	B3LYP			ωB97XD		
Ta	LanL2DZ	LanL2DZ	CEP-121G	LanL2DZ	LanL2DZ	LanL2TZ(f)
H	LanL2DZ	6-31++G**	CEP-121G	6-31++G**	6-311++G**	6-311++G**
*R*(H-H)/Å	0.876	0.881	0.827	0.894	0.910	0.920

**Table 2:** The distances between adsorbed hydrogen atoms *R*(H-H), adsorption enthalpy (Δ*H*), and interaction Gibbs energy values at 298 K (Δ*G*) obtained from structural optimization calculations of three dihydrogen complexes (see text) using ωB97XD and M06-2X (for the Mn complex).

	Ta (ωB97XD)	Cr (ωB97XD)	Mn (ωB97XD)	Mn (M06-2X)
*R*(H-H)/Å	0.920	0.820	0.828	0.795
Δ*H*/kJ mol^−1^	−105.5	−58.77	−40.43	−31.01
Δ*G*/kJ mol^−1^	−75.41	−48.58	−4.51	1.34

**Table 3:** Comparison of Δ*H* (interaction enthalpy at 0 K, including zero-point vibration) and the minimum AFIR energy required for dissociation, calculated for Mn1 and Mn2 (in kJ/mol) using the ωB97XD and M06-2X functionals. (The AFIR energy is the minimum positive energy barrier for the dissociation process, while Δ*H* is the negative enthalpy change during the adsorption process.)

Metal Complex	Experimental Δ*H*	Δ*H* (Interaction Enthalpy)		AFIR Energy (Minimum Required) *	
		ωB97XD	M06-2X	ωB97XD	M06-2X
Mn1	−50.2 [30]	−40.43	−31.01	70.0	42.5
Mn2	−27.0 [31]	−36.61	−25.45	67.5	47.5

* The energy required for dissociation was determined using AFIR calculations in increments of −2.5 kJ/mol. This increment is a numerical parameter chosen to control the precision of the barrier search and does not represent a physical quantity.

**Table 4:** Comparison of vibration entropy values (kJ/mol).

Metal Complex	DFT Functional	Temperature			
		150 K	225 K	298.15 K	450 K
Mn1(H_2_)	B3LYP	−0.005485945	−0.002297268	0.001528842	0.009773615
	CAM-B3LYP	0.033999387	0.036998491	0.040628389	0.04857012
	ωB97XD	−0.016341873	−0.015162207	−0.008367395	−0.006149344
	M06-2X	0.000998444	0.004596116	0.00465242	0.017907097
Mn2(H_2_)	B3LYP	0.005166585	0.009028706	0.013200066	0.021834367
	CAM-B3LYP	−0.009276537	−0.00591115	0.000302967	0.00613735
	ωB97XD	0.004123663	0.007438724	0.010313211	0.019277324
	M06-2X	−0.013554963	−0.010849453	−0.00366404	0.001581982
Mn1(D_2_)	B3LYP	−0.005485993	−0.002297326	0.001528787	0.009773529
	CAM-B3LYP	0.037163291	0.042399851	0.047748711	0.058250674
	ωB97XD	−0.013743185	−0.010264334	−0.001700778	0.003230651
	M06-2X	0.005093273	0.011008126	0.0127761	0.028582338
Mn2(D_2_)	B3LYP	0.005166693	0.009028758	0.013200197	0.021834477
	CAM-B3LYP	−0.005624175	−0.000246876	0.009335215	0.015967685
	ωB97XD	0.007680646	0.012969728	0.01742259	0.028986098
	M06-2X	−0.013554963	−0.004805395	0.004273374	0.012191903

**Table 5:** Comparison of isotope separation coefficient (*K*_D2_/*K*_H2_) for Mn complexes calculated with different DFT functionals.

Metal Complex	DFT Functional	Temperature			
		150 K	225 K	298.15 K	450 K
Mn1	B3LYP	22.63	5.44	2.84	1.60
	CAM-B3LYP	29.37	6.37	3.19	1.70
	ωB97XD	43.22	8.06	3.76	1.87
	M06-2X	17.79	4.64	2.55	1.53
	Experiment [30]	-	-	1.96 (313 K)–1.58 (363 K)	
Mn2	B3LYP	10.56	2.60	2.75	0.79
	CAM-B3LYP	13.09	2.99	1.53	0.83
	ωB97XD	26.71	6.04	3.08	1.66
	M06-2X	15.03	4.23	2.40	1.48
	Experiment [31]	4.2 (213 K)–2.2 (273 K)			-

## Data Availability

The original contributions presented in this study are included in the article/Supplementary Materials. Further inquiries can be directed to the corresponding author.

## Supplementary Materials

The following supporting information can be downloaded at: https://www.mdpi.com/article/10.3390/molecules31040636/s1. Figure S1: Two different views of the structure of [Mn(CO)(dppe)_2_-H_2_]+ (referred to as Mn1, and dppe = 1,2-bis(diphenylphosphino)ethane); Figure S2: Two different views of the structure of [Mn(CO)_3_(PCy_3_)_2_-H_2_]+ (referred to as Mn2); Table S1: Calculated Gibbs energy (ΔG in kJ/mol) at various temperatures for H_2_ and D_2_ adsorption on Mn1 and Mn2 complexes using density functional theory (DFT).
