# Supplementary material for: Quantum Chemical Study on the Temperature Dependence of Separation of Molecular Hydrogen and Deuterium Using Adsorption on Mn Dihydrogen Complexes
Source: Molecules. 2026 Feb 12;31(4):636. doi: 10.3390/molecules31040636 (PMC12942691; doi:10.3390/molecules31040636)
Supplement: Supplementary file 1 [file molecules-31-00636-s001.zip › molecules-3836298-supplementary.pdf]

Quantum chemical study on the temperature dependence of separation of molecular hydrogen and deuterium using adsorption on Mn dihydrogen complexes

Hao Xue, Naoki Kishimoto, and Shinya Takaishi

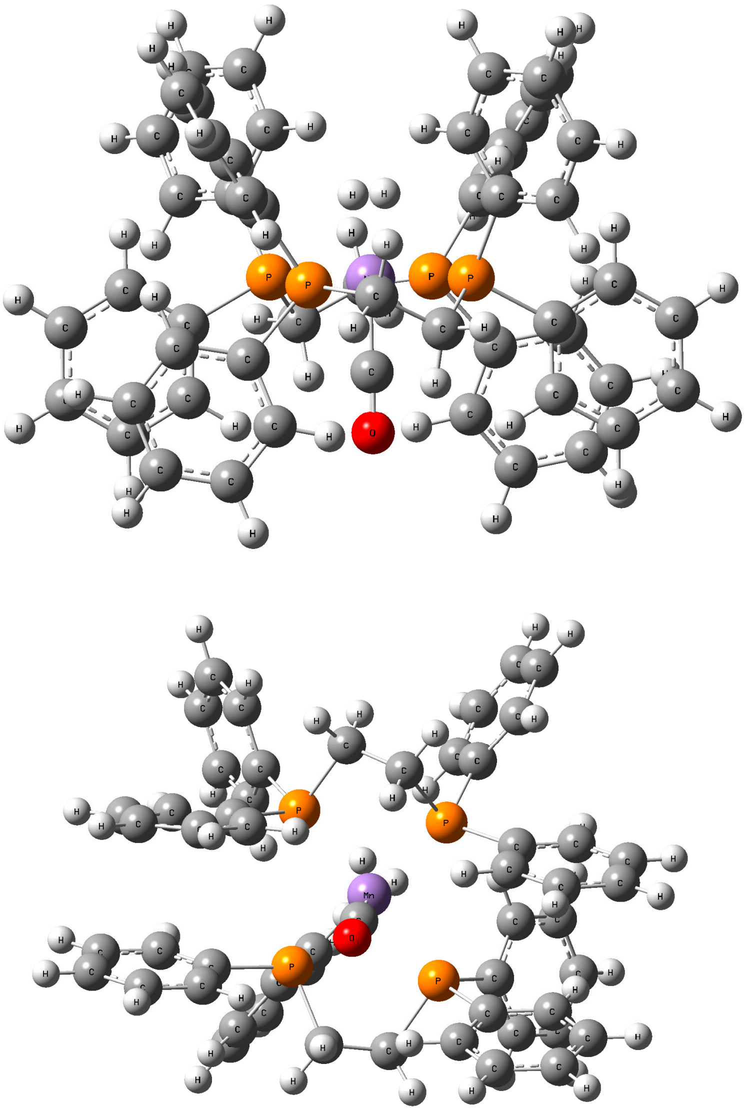

Figure S1. Two different views of the structure of $[Mn(CO)(dppe)_2\text{-}H_2]^+$ (referred to as Mn1, and dppe = 1,2-bis(diphenylphosphino)ethane).

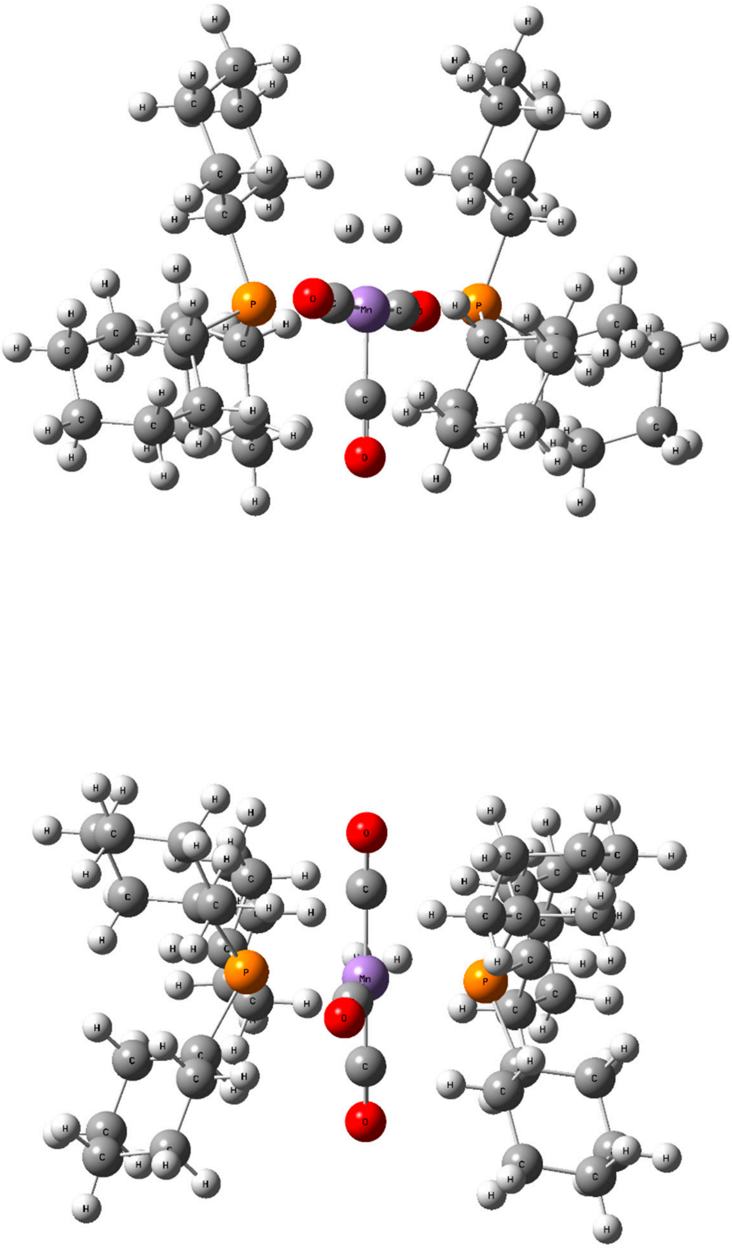

Figure S2. Two different views of the structure of $[Mn(CO)_3(PCy_3)_2\text{-}H_2]^+$ (referred to as Mn2).

Table S1. Calculated Gibbs energy (ΔG in kJ/mol) at various temperatures for H_2 and D_2 adsorption on Mn1 and Mn2 complexes using density functional theory (DFT).

Metal Complex	DFT functional	Molecule	Temperature				
			**0 K**	**150 K**	**225 K**	**298.15 K**	**450 K**
Mn1	B3LYP	H_2	-32.77	-19.77	-10.72	-2.21	18.45
		D_2	-38.62	-23.66	-13.89	-4.80	16.68
	CAM-B3LYP	H_2	-43.24	-36.44	-30.34	-23.94	-9.93
		D_2	-49.45	-40.66	-33.81	-26.82	-11.92
	ωB97XD	H_2	-40.43	-25.55	-15.17	-4.51	18.58
		D_2	-47.15	-30.24	-19.08	-7.79	16.24
	M06-2X	H_2	-31.01	-17.19	-7.54	1.34	23.49
		D_2	-36.58	-20.78	-10.41	-0.98	21.90
Mn2	B3LYP	H_2	-28.65	-17.14	-8.92	1.88	17.60
		D_2	-35.38	-20.08	-10.71	-0.63	18.50
	CAM-B3LYP	H_2	-40.53	-26.99	-17.66	-8.14	12.34
		D_2	-46.52	-30.20	-19.71	-9.19	13.02
	ωB97XD	H_2	-36.61	-24.80	-16.49	-7.95	10.50
		D_2	-42.63	-28.90	-19.86	-10.73	8.60
	M06-2X	H_2	-25.45	-12.75	-4.22	5.77	23.19
		D_2	-29.98	-16.13	-6.93	3.60	21.73
